# White rot fungi can be a promising tool for removal of bisphenol A, bisphenol S, and nonylphenol from wastewater

**DOI:** 10.1007/s11356-020-10382-2

**Published:** 2020-08-15

**Authors:** Agnieszka Grelska, Magdalena Noszczyńska

**Affiliations:** Institute of Biology, Biotechnology and Environmental Protection, Faculty of Natural Sciences, University of Silesia in Katowice, Jagiellońska 28, 40-032 Katowice, Poland

**Keywords:** White rot fungi, Endocrine-disrupting chemicals, Lignin-modifying enzymes

## Abstract

Endocrine-disrupting chemicals (EDC) are a wide group of chemicals that interfere with the endocrine system. Their similarity to natural steroid hormones makes them able to attach to hormone receptors, thereby causing unfavorable health effects. Among EDC, bisphenol A (BPA), bisphenol S (BPS), and nonylphenol (NP) seem to be particularly harmful. As the industry is experiencing rapid expansion, BPA, BPS, and NP are being produced in growing amounts, generating considerable environmental pollution. White rot fungi (WRF) are an economical, ecologically friendly, and socially acceptable way to remove EDC contamination from ecosystems. WRF secrete extracellular ligninolytic enzymes such as laccase, manganese peroxidase, lignin peroxidase, and versatile peroxidase, involved in lignin deterioration. Owing to the broad substrate specificity of these enzymes, they are able to remove numerous xenobiotics, including EDC. Therefore, WRF seem to be a promising tool in the abovementioned EDC elimination during wastewater treatment processes. Here, we review WRF application for this EDC removal from wastewater and indicate several strengths and limitations of such methods.

## Introduction

The past two decades have seen growing awareness of the possible adverse effects on human and animal health resulting from exposure to endocrine-disrupting chemicals (EDC). This group includes xenoestrogens, i.e., exogenous substances with estrogen activity, to which bisphenol A (BPA), bisphenol S (BPS), and nonylphenol (NP) belong (Pothitou and Voutsa 2008; Michałowicz 2014; Pookpoosa et al. 2014; Garcia-Morales et al. 2015; Guo et al. 2016; Catanese and Vandenberg 2017; Yan et al. 2017; Diao et al. 2017; Urriola-Muñoz et al. 2017; Česen et al. 2018; Wu et al. 2018b; Noszczyńska and Piotrowska-Seget 2018). EDC are associated with a wide variety of disorders (Ben-Jonathan 2004; Kandaraki et al. 2011; Schug et al. 2011; De Coster and Van Larebeke 2012). Despite the negative effects of EDC, they are widely used in industry (Noszczyńska and Piotrowska-Seget 2018; Rodríguez-Peña et al. 2019). As a result of the extensive production, processing, and transport of EDC-containing products and EDC themselves, these compounds often contaminate aquatic environments, as shown in Table 1 (Pothitou and Voutsa 2008; Terzić et al. 2008; Janex-Habibi et al. 2009; Kasprzyk-Hordern et al. 2009; Martin Ruel et al. 2010; Rosal et al. 2010; Yu et al. 2013; Yang et al. 2014a, b; Jin and Zhu 2016; Lu et al. 2019; Radwan et al. 2020; Singh and Thakur 2020). Currently, wastewater treatment systems are not able to cope with EDC removal, which are present in wastewater in trace amounts even at ng L^−1^ (Niemuth and Klaper 2015; Bai and Acharya 2019; Lv et al. 2019). In response to this problem, various techniques of degradation, transformation, and/or removal of EDC from wastewater have been applied. Among them, white rot fungi (WRF) seem to be an efficient and ecologically friendly method with the potential to transform most of the xenobiotics. The majority of prior research has been conducted on EDC removal by WRF. Our aim is to summarize the existing knowledge and indicate gaps in the research that need to be filled.

**Table 1:** An overview showing detectable EDC concentrations at various aquatic environments

EDC	Type of water reservoir	Concentration [ng L^−1^]	Location	References
Bisphenol S	Surface water	8.9	Liaohe River, China	Jin and Zhu (2016)
	Surface water	6.4	Taihu Lake, China	Liu et al. (2017)
	Surface water	0.29–18.99	Hangzhou Bay, China	Yang et al. (2014a)
	Wastewater	23.6–31.2	Albany, New York	Xue and Kannan (2019)
	Surface water	15–3640	Cooum River, India	Yamazaki et al. (2015)
Bisphenol A	Surface water	29	Liaohe River, China	Jin and Zhu (2016)
	Surface water	23	Taihu Lake, China	Liu et al. (2017)
	Wastewater	70–1680	Quebec, Canada	Mohapatra et al. (2011)
	Surface water	55–162	Aisonas River, Greece	Stasinakis et al. (2012)
	Groundwater	79	Europe	Loos et al. (2010)
Nonylphenol	Surface water	34.4–86.6	Beijing, China	Wang et al. (2015)
	Groundwater	3.4–41.5	Beijing, China	Wang et al. (2015)
	Wastewater	24–70.4	Beijing, China	Wang et al. (2015)
	Surface water	558–2704	Aisonas river, Greece	Stasinakis et al. (2012)
	Groundwater	83	Europe	Loos et al. (2010)

## Endocrine-disrupting chemicals

EDC are nonpersistent or persistent chemicals (Cajthaml 2015; Corrales et al. 2015). Nonpersistent EDC include chemicals that are rapidly degraded in the environment and are quickly metabolized in and eliminated from the human body (Nelson et al. 2020). Persistent EDC are stable in both the environment and the human body and undergo significant biomagnification for a short or long period (Song et al. 2014; de Voogt 2018). EDC are described as chemically synthesized or naturally existing compounds, absent within living organisms, that interfere with the endocrine system by imitating or inhibiting endogenous hormones, thus consecutively inducing hormonal dysfunctions, having a negative impact on living organisms (S. Environ. Prot. Agency 1997; Gore et al. 2015; Björnsdotter et al. 2017; Lauretta et al. 2019). On the one hand, EDC may show an affinity to specific nuclear receptors known as peroxisome proliferator-activated receptors (PPARs) (Cocci et al. 2013; Agarwal et al. 2017; Sharma et al. 2018). PPARs are normally involved in the binding of certain ligands such as steroid hormone molecules or fatty acids, acting as transcription factors, thus regulating the expression of genes associated with lipid metabolism in the organism (Urriola-Muñoz et al. 2014; Catanese and Vandenberg 2017; Gupta and Pushkala 2019). Therefore, the influence of EDC on PPARs contributes to an elevated adipocyte level in the body and the risk of obesity (Heindel et al. 2015; Ahn et al. 2020). On the other hand, the main targets of EDC are estrogenic receptors (ERα and ERβ), which can be stimulated or inactivated by appropriate conjunction of the ligand. Thus, EDC work either as antagonists or agonists of ERs, disrupting the estrogenic balance in organisms (Rogers et al. 2013; Sifakis et al. 2017). BPA and NP are among the best known xenoestrogens. However, due to the increasing use of BPS in industry and its widely demonstrated negative impact on human hormonal system, in the scientific literature, more and more attention is devoted to this compound (Viñas and Watson 2013; Catanese and Vandenberg 2017; Urriola-Muñoz et al. 2017; Qiu et al. 2018; Gupta and Pushkala 2019).

Bisphenol A has become one of the most intensively manufactured chemicals in the world due to demonstrating the finest properties for plastic production (Noszczyńska and Piotrowska-Seget 2018). Numerous studies have investigated BPA effects on the human body and animals (Zhu et al. 2015; Quesada et al. 2002; Braun et al. 2009; Izzotti et al. 2009; Pfeifer et al. 2015; Leung et al. 2017; Maćczak et al. 2017; Pinney et al. 2017; Tian et al. 2018; Grandin et al. 2019; Özel et al. 2019; Gao et al. 2020; Rasdi et al. 2020; Tassinari et al. 2020; Wu and Seebacher 2020; Wu et al. 2020a; Pan et al. 2020). Since BPA has a comparable structure to that of natural estrogen 17β-estradiol, it can bind to ERα and ERβ, though with 1000-fold less affiliation than estradiol (Gray et al. 2004; vom Saal and Hughes 2005; Takayanagi et al. 2006; Gray et al. 2004; vom Saal and Hughes 2005; Takayanagi et al. 2006). Despite this, BPA, even at low doses measured in ng L^−1^, is capable of disrupting human cell function by interacting with extranuclear receptors (Michałowicz 2014). For instance, BPA binds to membrane estrogen receptors and GPR30 protein-coupled receptors and, hence, participates in nongenomic pathways (Rubin 2011; Cygankiewicz et al. 2015). The literature review shows that BPA is not only an endocrine-disrupting chemical, but it also causes damage to hepatocytes through oxidative stress (Kourouma et al. 2015; Elswefy et al. 2016; Li et al. 2017). BPA can modulate the immune response, has mutagenic activity toward eukaryotic cells, and leads to obesity (Michałowicz 2014; Wu et al. 2020b). Moreover, BPA disrupts microtubule organization and centrosome function, hence showing the vast spectrum of cancer-promoting effects, including induction of prostate and mammary cancer formation (Seachrist et al. 2016; Ho et al. 2017; Mesnage et al. 2017). In addition, since fetuses, infants, and young children do not possess feedback to regulate the synthesis, activity, and elimination of hormones, BPA is particularly harmful to them (Rykowska and Wasiak 2006; Braun et al. 2009). Exposing children to BPA may result in higher levels of inattention, anxiety, hyperactivity, depression, and behavioral problems (Ejaredar et al. 2017; Wiersielis et al. 2020). In the European Union and the USA, the use of BPA-based polycarbonate bottles for feeding babies was banned. In France, the use of BPA in all packaging containers and dishes planned to come into direct contact with food was prohibited (Euroactiv 2015). In Denmark and Belgium, BPA was not allowed for the production of food contact materials and articles for children under 3 years (Services-Global MT 2013).

Due to the deleterious effects of BPA on human health resulting in tightened provisions on BPA in the abovementioned regions, alternative bisphenol compounds have been used for industrial applications (Héliès-Toussaint et al. 2014; Chen et al. 2016). Among them, bisphenol S is increasingly applied as a substitute for BPA (Wu et al. 2018a). Presently, BPS is delivered worldwide at the level of 10,000–100,000 t annually (ECHA 2020). As a result of intensive manufacturing and poor biodegradability, BPS presence was discovered in surface water in the amount of 0.22–52 ng L^−1^ in 2013, which by 2016 had already reached 16–410 ng L^−1^ (Lake Taihu in China) (Jin and Zhu 2016; Liu et al. 2017). Although the affinity of BPS to ERs is 100,000-fold lower than that of 17β-estradiol and 37 times weaker than BPA, BPS is able to bind to these receptors, thus causing their activation, changing the hormone levels and the expression of genes controlled by ERα and ERβ (Klopman and Chakravarti 2003; Grignard et al. 2012). Additionally, BPS functions as a weak androgen receptor (AR) agonist (Molina-Molina et al. 2013; Zenata et al. 2017). A number of authors have demonstrated in laboratory studies that BPS beyond endocrine disruptive activity is cytotoxic, immunotoxic, neurotoxic, and genotoxic (Peyre et al. 2014; Rochester and Bolden 2015; Feng et al. 2016; Zhang et al. 2016; Dong et al. 2018; Qiu et al. 2018; Mas et al. 2020). Due to the disruption of centrosome function and microtubule organization, BPS such as BPA exerts a vast spectrum of cancer-promoting effects, but it incites a stronger reproductive and DNA damage response than BPA (Ho et al. 2017; Deng et al. 2018; Lin et al. 2019; Song et al. 2019).

Nonylphenol is a chemical member of the alkylphenol group (Chokwe et al. 2017). NP is composed of a phenyl group joined to a nine-carbon lipophilic chain. The varied structure provides it both hydrophilic and hydrophobic character; hence, it acts as an effective uncharged surfactant (John et al. 2000; Soares et al. 2008). Therefore, NP is a suitable raw material in the production of paints, cosmetics, detergents, hair dyes, and pesticides. In addition, the presence of NP is observed in vinyl chloride (PVC), which can contaminate water passing through PVC plumbing (EPA 2005). Due to its high hydrophobicity, resistance to biodegradation, and low solubility, it is prone to accumulate in various environmental matrices (Krupiński and Długoński 2011). Consequently, NP was detected in water averaging 0.805 μg L^−1^ in China; 12.61 μg L^−1^, 12.2 μg L^−1^, and 6.08 μg L^−1^ in recreational water, wastewater discharges, and drinking water, respectively, in Mexico; 1.6 μg L^−1^ in Japan; and 0.22 μg L^−1^ in Ukraine (Hoai et al. 2003; Zhang et al. 2017; Vystavna et al. 2018; Vargas-Berrones et al. 2020). However, as evaluated by the Water Framework Directive of the European Union, the maximum NP concentration in water in Europe is 2 μg L^−1^ (EU, Directive 2013/39/EU 2013), while in the USA, the Environmental Protection Agency (EPA U 2010) establishes this dose as 6.6 μg L^−1^ (EPA 2005). Owing to NP’s lipophilic properties, it can be deposited in adipose tissue (Yu et al. 2020). Also, NP is capable of binding to ER receptors by competing with natural estrogen (E2), although with lower affinity than the natural hormone (Noorimotlagh et al. 2017). As a result of the above mechanism, NP induces disorders in men, including a reduction in the level of circulating testosterone in the blood, decreased activity of antioxidant enzymes in sperm, and disturbed testicular structure as well as enhanced apoptosis of Sertoli cells (Cardinali et al. 2004; Gong et al. 2009; Aly et al. 2012; Hu et al. 2014; Urriola-Muñoz et al. 2014). On the other hand, a study showed that high exposure to NP of women in the second trimester of pregnancy led to reduced birth weight of the child and shortened the gestational age (Chang et al. 2013).

## White rot fungi

In the forest ecosystem, wood decomposition is a key process in the carbon and nutrient cycle (Purahong et al. 2016). The rate of wood decay is determined by external factors such as substrate quality and climate as well as the diversity and activity of the organisms that contribute to degradation (Brischke et al. 2006). Moreover, wood contains a high lignin content, which significantly hinders the breakdown process (Purahong et al. 2016). WRF are among the best lignin degradants. Their name derives from a specific process of bleaching which occurs during the degradation of wood by fungi (Ten Have and Teunissen 2001). Interestingly, it was demonstrated that Fe_3_O_4_ nanomaterials combined with the WRF *Phanerochaete chrysosporium* have promising potential for application in lignocellulose degradation (Huang et al. 2019). WRF are primarily classified as *Basidiomycota* type; however, also a limited number represent *Ascomycota* (Patel et al. 2014). These fungi are common in nature, usually found in forest ecosystems, more often in deciduous than coniferous forests (Singh and Singh 2014). Besides a capacity for lignin degradation, WRF have remarkable versatility in breaking down a wide variety of complex and resistant environmental contaminants that pollute aquatic ecosystems, posing a potential threat to human and animal health. It is quite well proven that WRF have a biochemical ability to degrade sulfonamide antibiotics and important categories of toxic, organic xenobiotics such as polycyclic aromatic hydrocarbons (PAH), 1,1,1-trichloro-2,2-bis(4-chlorophenyl) ethane (DDT), synthetic textile dyes, polychlorinated biphenyls (PCB), pentachlorophenols (PCP), and trinitrotoluene (TNT). Furthermore, these organisms are capable of heavy metal immobilization via unique extracellular oxidative enzyme systems, extracellular chelation with organic acids, cell wall cation exchange, and intracellular bioaccumulation (Ellouze and Sayadi 2016; Kachlishvili et al. 2016; Stella et al. 2017; Guo et al. 2018; Vršanská et al. 2018; Lee et al. 2020; Xiao and Kondo 2020). In addition to these xenobiotics, more and more publications demonstrate the significant potential of WRF to break down EDC, especially BPA and NP (Hirano et al. 2000; Saito et al. 2004; Lee et al. 2005; Soares et al. 2005; Cabana et al. 2007a; Shin et al. 2007; Cajthaml et al. 2009; Hofmann and Schlosser 2016; Llorca et al. 2017; Pezzella et al. 2017; Křesinová et al. 2018; Zdarta et al. 2018). Knowledge about the decomposition of BPS by WRF is sparse (Zdarta et al. 2018). However, due to the increasing usage of BPS and its harmful influence on human beings, this gap should be filled.

WRF, especially water-adapted ones, should be considered for the removal of EDC such as in specifically designed treatment modules for wastewater. WRF for the degradation of organic EDC use the same mechanisms that are involved in ligninolysis (Pointing 2001). These mechanisms involve a number of extracellular, broad-acting lignin-modifying enzymes (LMEs). LMEs, besides intracellular and mycelium-related enzymes, might also catalyze biosorption of EDC to whole-cell WRF which may be the first biodegradation stage of these chemicals (Harms et al. 2011).

## Lignin-modifying enzymes

WRF produce lignin-modifying enzymes, which, apart from their ability to degrade lignin, are active against xenobiotics, including EDC (Hashim et al. 2018). There are four main classes of LMEs: laccases, manganese peroxidases, lignin peroxidases, and versatile peroxidases (Cabana et al. 2007b; Cajthaml 2015). Although WRF are capable of producing all classes of enzymes, particular strains may not release all of them together (Yang et al. 2013a). LMEs are synthesized by fungi undergoing secondary metabolism, as lignin oxidation does not supply energy to them. The limited nutrient quantity in the medium, such as carbon or nitrogen, as well as hypoxia stimulates the synthesis of these enzymes (Niku-Paavola et al. 1990; Pointing 2001; Marco-Urrea et al. 2010; Mattila et al. 2020). Mixing of liquid fungi cultures generates laccase production but inhibits the synthesis of lignin and manganese peroxidase. On the other hand, high oxygen molecular pressure leads to increased secretion of lignin and manganese peroxidase. Frequently, several LME isoforms are produced by fungi depending on the fungus strain and culture conditions (Torres et al. 2003; Wesenberg et al. 2003; Levin et al. 2004; Yang et al. 2013b; Kinnunen et al. 2016). Temperature, pH, agitation, or the presence of inorganic salts or heavy metals affects the breakdown of endocrine-disrupting chemicals by LMEs. These parameters influence the activity of enzymes, their stability, and substrate specificity (Kim and Nicell 2006; Soares et al. 2006; Auriol et al. 2007; Kinnunen et al. 2016). The advantage of fungi over bacteria in lignin mineralization results from the production and secretion of LMEs outside the cell. In addition, fungi can operate in a wide range of temperatures and pH values, while enzymes are synthesized during nutrient deficiency (Robinson et al. 2001; Arora and Gill 2005; Urek and Pazarlioglu 2007; Dhakar and Pandey 2013; Hariharan and Nambisan 2013). Expanding fungal hyphae also make it possible to reach contaminants inaccessible to bacteria (Cabana et al. 2007b). Moreover, WRF enzymes are nonspecific so that the fungi can transform compounds resembling lignin in their chemical structure. Such compounds may include pesticides, alkanes, aromatic hydrocarbons, or bisphenol A (Harms et al. 2011). The secretion of LMEs outside the cell gives fungi access to nonpolar and insoluble substances (Llorca et al. 2017). Meanwhile, the presence of functional groups such as amine, hydroxyl, or alkyl groups in chemical compounds, acting as electron donors, makes these compounds more susceptible to electrophilic oxygenase attack. Therefore, WRF effectively remove phenolic compounds such as BPA and NP (Tadkaew et al. 2011; Yang et al. 2013b).

## Manganese peroxidase

The peroxidase most frequently produced by WRF is manganese peroxidase (MnP). MnP is a glycoprotein containing a prosthetic group in the form of a heme molecule (an iron complex with protoporphyrin IX). There are existing multiple MnP isoforms with a molecular weight between 32 and 62.5 kDa (Qiu et al. 2019). This enzyme was discovered for the first time in *P. chrysosporium* almost 30 years ago, and it is the only heme peroxidase with a single-electron mechanism of Mn^2+^ oxidation reaction (Pollegioni et al. 2015). MnP catalyzes the oxidation of Mn^2+^ to Mn^3+^ via hydrogen peroxide (H_2_O_2_) required as an electron acceptor (Dashtban et al. 2010).

$$ 2{\mathrm{Mn}}^{2+}+2{\mathrm{H}}^{+}+{\mathrm{H}}_2{\mathrm{O}}_2\to 2{\mathrm{Mn}}^{3+}+2{\mathrm{H}}_2\mathrm{O} $$

**Fig. 1:**
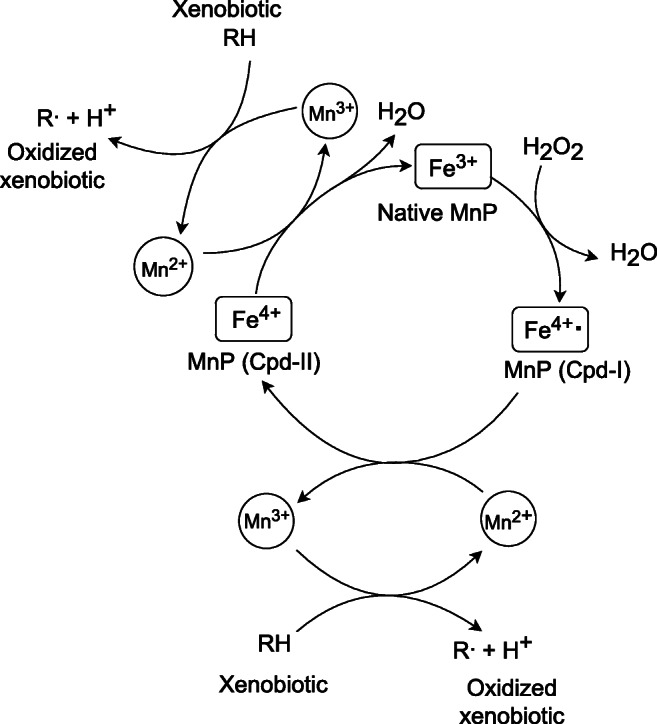
MnP catalytic cycle (Pollegioni et al. 2015, modified)

The reaction catalyzed by MnP begins with the conversion of the native enzyme through hydrogen peroxide to the first transitional compound [Cpd-I], which constitutes the Fe^4+^ radical complex (Fig. 1) (Manavalan et al. 2015). At the same time, the Mn^2+^ ion is oxidized to Mn^3+^, and a second transitional compound [Cpd-II] is formed. Mn^3+^ ion is then separated from the surface of the enzyme and is linked to carboxylic acids, in particular, oxalate and malate. The chelated Mn^3+^ complex acts as an oxidant of phenolic rings, reducing to the Mn^2+^ ion and producing a transitional phenoxyl radical, resulting in the formation of various breakdown products (Pollegioni et al. 2015). The native enzyme is created from the Cpd-II, through electron release and oxidation of Mn^2+^ to the Mn^3+^ complex. The chelated Mn^+3^ can restore the phenoxyl radical, which oxidizes sequential phenolic rings (Manavalan et al. 2015). The Mn^3+^ complex is restricted exclusively to the oxidation of phenolic compounds such as simple phenols, amines, dyes, and lignin phenolic compounds. In relation to nonphenolic compounds, the complex remains inactive due to deficient redox potential (Manavalan et al. 2015; Żygo and Prochoń 2017). Besides, the action of MnP is entirely inhibited by inhibitors such as Hg^2+^, Pb^2+^, Ag^+^, NaN_3_, lactate, or ascorbic acid (Manavalan et al. 2015).

## Lignin peroxidase

Lignin peroxidase (LiP) is a glycoprotein with a molecular weight between 38 and 46 kDa, which contains heme as a prosthetic group, whereas the entire enzyme is stabilized via 4 disulfide bridges. The LiP structure is very akin to MnP since it is a globular protein composed of 11–12 α-helixes containing the central cavity with a heme group (Manavalan et al. 2015; Pollegioni et al. 2015). Such a considerable analogy of both enzymes may point to divergent selection (Pollegioni et al. 2015). Despite the structural resemblance, LiP exhibits significantly greater redox potential [*E*_0_*′* ~ 1.2 V] in comparison with MnP [~ 0.8 V], due to a higher deficit of ferrous atom electrons in the porphyrin ring (Abdel-Hamid et al. 2013; Pollegioni et al. 2015). This advantage allows LiP to oxidize, along with phenolic compounds, even nonphenolic xenobiotics and lignin components, regardless of the presence of a mediator. Nevertheless, an elevated concentration of hydrogen peroxide or compounds such as acetone and diethyl ether as well as dioxane functions as LiP inactivators in many fungi (Manavalan et al. 2015).

**Fig. 2:**
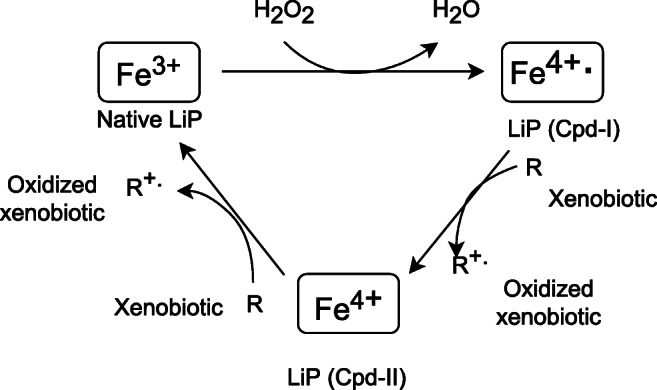
LiP catalytic cycle during degradation of xenobiotics (Abdel-Hamid et al. 2013, modified)

**Fig. 3:**
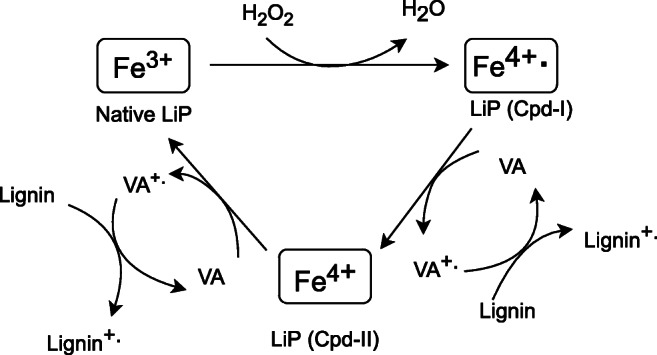
LiP catalytic cycle during lignin degradation using VA as an electron donor (Abdel-Hamid et al. 2013, modified)

LiP disintegrates lignin and xenobiotics in three stages, involving hydrogen peroxide (Fig. 2) (Pollegioni et al. 2015). The catalytic reaction is initiated by oxidation of the native LiP enzyme to the transient compound [Cpd-I], which forms the radical complex Fe^4+^. Crucial in this reaction is H_2_O_2_, serving as an electron acceptor. In a further stage, the transitional compound [Cpd-I] is reduced by a xenobiotic such as EDC to a second transitional compound [Cpd-II] (Abdel-Hamid et al. 2013; Falade et al. 2017). Simultaneously, the xenobiotic molecule converts into a radical form through electron depletion, followed by nonenzymatic reactions leading to the formation of the final degradation product (Dashtban et al. 2010). In order to complete the enzymatic cycle and regain the native form, LiP must be reduced anew, with the consequent occurrence of the subsequent xenobiotic radical (Abdel-Hamid et al. 2013). Concerning lignin decomposition, LiP favors veratryl alcohol (VA) as a nonphenolic substrate providing electrons for redox reactions. As a natural metabolite of fungi in contact with lignin, VP increases the catalytic properties of the enzyme and the velocity of lignin breakdown (Muszyńska et al. 2017). As a result of VA oxidation, a radical cation of this compound is formed and acts as a direct lignin oxidant (Fig. 3) (Abdel-Hamid et al. 2013).

## Versatile peroxidase

Similar to previous peroxidases, versatile peroxidase (VP) also presents a glycoprotein structure with a molecular weight varying between 38 and 45 kDa, with heme in the central region, functioning as an enzyme cofactor. VP has been originally reported in the *Pleurotus eryngii* species, whereas at this point, the presence of VP has been only confirmed in the species of *Pleurotus* and *Bjerkandera* fungi (Abdel-Hamid et al. 2013). The versatility of this peroxidase is achieved by combining the catalytic properties of MnP and LiP, through the ability to oxidize Mn^+2^ and due to high redox potential (Abdel-Hamid et al. 2013). Hence, VP is able to degrade both nonphenolic and phenolic components of lignin and xenobiotics, as well as numerous dyes (e.g., Reactive Black 5—used for dyeing wool, cotton, and viscose) (Pollegioni et al. 2015). Moreover, a hybrid VP provides multiple binding sites for substrates. The catalytic efficiency of VP in the oxidation of Mn^+2^ ions is comparable to MnP. However, in the case of oxidation of phenolic and nonphenolic components of lignin, this enzyme is ten times less productive than LiP (Pollegioni et al. 2015).

**Fig. 4:**
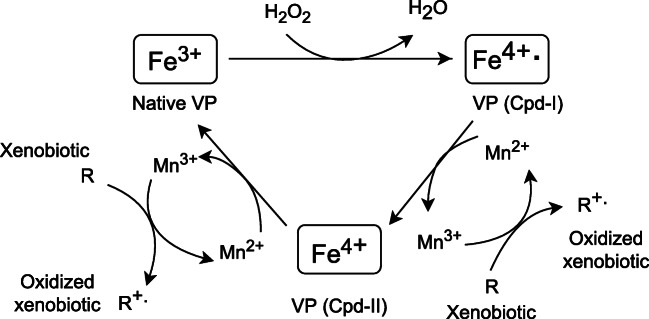
VP catalytic cycle during oxidation of phenolic compounds (Ravichandran and Sridhar 2016, modified)

The mechanism of phenolic compound breakdown by VP is analogous to MnP. At the first stage, the cofactor Fe^4+^ complex of the native enzyme is oxidized to the transient compound [Cpd-I] radical in the presence of H_2_O_2_ (Fig. 4). Simultaneously, Mn^2+^ is converted into Mn^3+^, and then the oxidized ion combines with carboxylic acids to maintain its stability (Pollegioni et al. 2015). The Mn^3+^ complex functions as an oxidant of phenolic compounds leading to the formation of a transient phenoxyl radical and, consequently, to the generation of final breakdown products (Manavalan et al. 2015). As a result of manganese ion oxidation, a second transient compound [Cpd-II] is formed, which can revert to the initial enzyme form by gaining an electron. Electron loss allows the Mn^3+^ to oxidize subsequent phenolic rings (Manavalan et al. 2015).

**Fig. 5:**
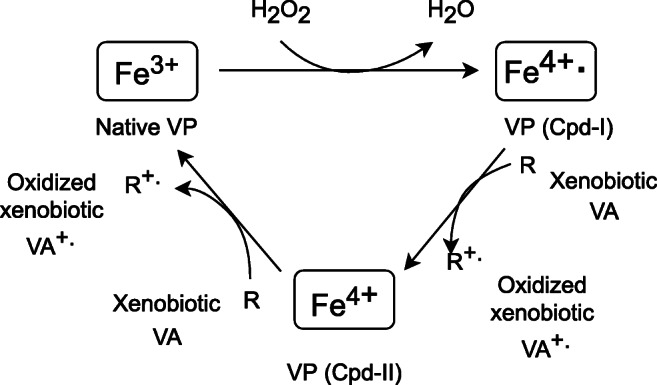
VP catalytic cycle during oxidation of VA and nonphenolic compounds (Abdel-Hamid et al. 2013, modified)

On the other hand, VP employs an identical mechanism as LiP for the elimination of both nonphenolic compounds and lignin polymer. The native enzyme is oxidized to a transient compound (Cpd-I) radical involving hydrogen peroxide. Cpd-I is further reduced by a single electron delivered from a nonphenolic compound (xenobiotic, VA) to a second transition compound (Cpd-II) (Fig. 5) (Abdel-Hamid et al. 2013; Falade et al. 2017). Hence, a radical form of xenobiotic molecule is created, which is exposed to nonenzymatic reactions (coupling, polymerization, side-chain splitting, demethylation, regrouping) (Dashtban et al. 2010). Termination of a cycle by VP is possible by continued reduction of the Cpd-II compound, as well as the simultaneous generation of a new nonphenolic radical molecule (Abdel-Hamid et al. 2013).

## Laccase

**Fig. 6:**
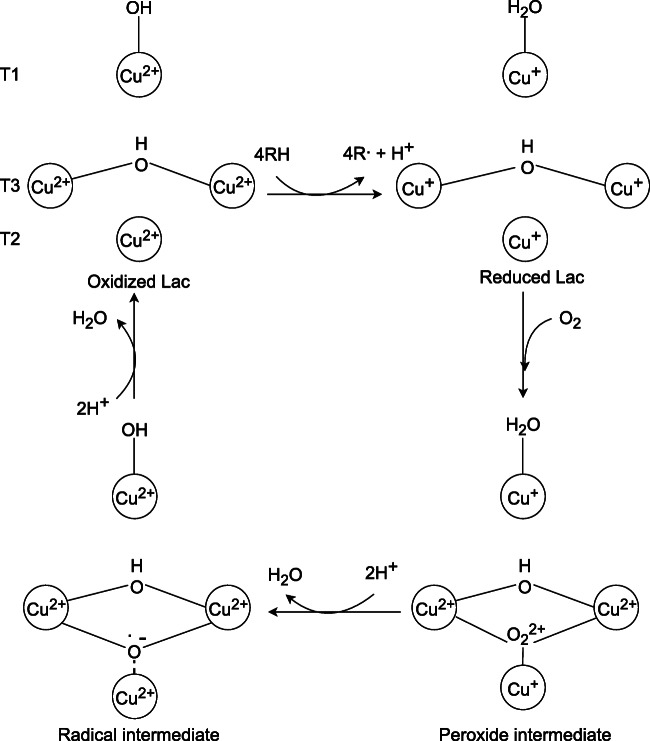
Lac catalytic cycle (Pollegioni et al. 2015, modified)

Laccase (Lac) is the most commonly occurring enzyme in the environment among LMEs. Lac has been primarily detected in the Asian tree *Toxicodendron vernicifluum* species. Currently, this enzyme is identified in numerous species of plants and microorganisms such as bacteria and fungi, including a majority of WRF (e.g., *P. eryngii*, *Trametes versicolor*, *P. chrysosporium*). Lac, together with the rest of LMEs, belongs to glycoproteins, although it has a greater molecular weight, reaching even up to 150 kDa, as well as a distinctive blue color. In the central region of the enzyme, 4 copper cations are located, divided into 3 types (Manavalan et al. 2015; Pollegioni et al. 2015). Type 1 (T1) exhibits a high level of absorption at 600 nm, which is responsible for the unique pigmentation of an enzyme. Copper type 2 (T2) is deprived of color, though it possesses paramagnetic properties, whereas type 3 (T3) is composed of two interconnected diamagnetic cations exhibiting peak absorbance equal to 330 nm (Strong and Claus 2011). Lac belongs to the oxidases group; therefore, it participates in the 4 electron transition from distinct substrate molecules to O_2_, which is subsequently reduced to H_2_O_2_ (Fig. 6) (Muszyńska et al. 2017).

The catalytic cycle of this enzyme is initiated by the progressive oxidation process of 4 separate substrate particles and simultaneously the passage of 4 subsequent electrons to the copper cations in the active center, resulting in a state of full Lac reduction (Pollegioni et al. 2015). In the second stage, a single O_2_ molecule joins the T3 and T4 copper cations, rapidly transforming into a transition peroxide by obtaining two individual electrons from both T3 ions. However, this condition does not persist long since oxygen falls apart into an oxyradical, engaging 2 additional electrons from copper molecules, which split the oxygen bonds. This is accompanied by the release of the first water particle. Completion of a Lac catalytic cycle is achieved by total oxidation of each of the four copper ions and release of a second water molecule (Pollegioni et al. 2015). The above reaction mechanism allows Lac to degrade phenols and phenolic components of lignin, as well as nonphenolic compounds, but only in the presence of redox mediators (Abdel-Hamid et al. 2013).

## Potential of WRF to remove BPA, BPS, and NP from wastewater

Due to the increasing urbanization, EDC are increasingly produced by many branches of industry. As a consequence, these substances penetrate the soil and water, which causes significant pollution affecting these ecosystems. Despite EDC being present in the environment mainly at low concentrations in the order of ng L^−1^, they can be a serious threat both for aquatic animals and humans (Solé and Schlosser 2015). Therefore, such recalcitrant compounds have to be removed from wastewater. Since traditional sewage treatment plants using activated sludge processes eliminate EDC only to a limited extent, there is a need to look for other effective methods for their removal (Ahmed et al. 2017; Cecconet et al. 2017). Numerous attempts, including adsorption, filtration, chlorination, coagulation/flocculation, Fenton/photo-Fenton degradation, sonochemical degradation, photochemical/photocatalytic oxidation, ozonation, and hybrid processes with physical and thermal approaches, have been made to remove EDC from water (Yoon et al. 2007; Sharma et al. 2009; Zhang and Li 2014; Ahmed et al. 2017). However, these procedures are costly and often result in equally toxic secondary impurities. Alternatively, the use of WRF for remediation of contaminated water is cost-effective and sustainable. WRF compared to other potential bioremediation bacteria are not adversely affected by the antibiotics commonly found in wastewater (Boer 2018). On the other hand, WRF need a second source of carbon, as the abovementioned EDC degradation takes place as part of the secondary metabolism. Despite that, in contrast to bacteria, WRF are able to decompose EDC even at low concentrations (Mir-Tutusaus et al. 2018). Many different studies have been conducted on the effectiveness of removing EDC from the environment (Kim et al. 2007; Toyama et al. 2009; Huang et al. 2014; Zhang and Li 2014; Zielińska et al. 2016; Csuros et al. 2018; Li et al. 2020; Oh et al. 2020; Stenholm et al. 2020; Suyamud et al. 2020; Zhang et al. 2020). Much of this research has been devoted to the use of both whole WRF cells and extracted enzymes in EDC degradation, although tests on the former were more repeatedly reported. As this review focused on BPA, BPS, and NP removal using WRF, therefore, in the description below, particular emphasis has been placed on the use of these organisms in the removal of the abovementioned compounds.

For research applications, WRF systems are constructed in the form of bioreactors, providing a constant substrate supply, thus maintaining controlled environmental conditions (Tadkaew et al. 2011; Ahmed et al. 2017). Examples of the efficiency of EDC removal by whole-cell WRF cultures are shown in Table 2. The results vary among studies since the degradation capacity depends on multiple factors such as the molecular structure of the xenobiotic, the species of the applied fungus, and the type of secreted enzymes.

**Table 2:** Removal efficiency (%) of various EDC achieved by different whole-cell WRF species under distinct culture conditions

WRF species	Culture conditions	EDC	Initial concentration (mg/L)	Incubation time	Removal efficiency (%)	References
*T. versicolor*	Bubble column/internal loop airlift bioreactor Temp 28 °C	Bisphenol A	22.83	8 days	100	Pezzella et al. (2017)
		Nonylphenol	22.04	8 days	84	
	Temp 28 °C	Bisphenol A	500	6 h	98.7	Brazkova (2019)
	Batch bioreactor Temp 25 °C pH = 4.5	Nonylphenol	0.0017	2 days	52.9	Llorca et al. (2017)
*P. ostreatus*	Continuous flow trickle-bed bioreactor Temp 28 °C	Bisphenol A	2	12 days	> 90	Křesinová et al. (2018)
		Nonylphenol	2	12 days	> 90	
*P. chrysosporium*	Bubble column/internal loop airlift bioreactor Temp 28 °C	Nonylphenol	22.04	8 days	65	Pezzella et al. (2017)
		Bisphenol A	22.83	8 days	60	

Several authors have shown that the first step of organic pollutant biodegradation by WRF may be sorption of these compounds to the fungal mycelium caused by the high surface to volume ratio of WRF (Zafar et al. 2007; He et al. 2010; Ding et al. 2013; Nguyen et al. 2014). On the other hand, it was revealed that crude or purified LME solutions were able to catalyze EDC biodegradation in the absence of sorption to fungal mycelium (Yang 2012). It results from the hydrophobic character of these compounds (log*k*_ow_ ≥ 3.2), which determines the adsorption behavior of EDCs (Krupadam et al. 2011). Most of the studies have mainly revealed EDC removal from the aqueous phase without monitoring the extent of biosorption (Pezzella et al. 2017; Mtibaà et al. 2018; Brazkova 2019). It creates difficulties in assessing the relative contribution of biosorption and biodegradation to the general removal of the highly hydrophobic EDC. Only a few studies on biosorption effects alone on EDC treatment have been performed. Among them are the studies performed by Nguyen et al. (2014) who observed not higher than 30% efficiency of BPA sorption to inactivated *T. versicolor* biomass, and Yonten et al. (2016) who gained up to 90% of BPA removal by adsorption to *Pleurotus eryngii* immobilized on polymeric resin. Immobilization greatly facilitates biosorption by increasing the mechanical strength of the biosorbent and reusability (Wu and Yu 2007). Additionally, factors such as pH or volume of the sample solution can influence the course of the sorption treatment. Increasing biosorption of BPA was observed in the pH range of 7–11, with maximum adsorption at pH 11, while a decreasing trend was noted at the lower pH of 2–7 (Yonten et al. 2016). The same authors also revealed that BPA is removed from the solution exponentially only up to a specific moment, followed by a constant value, due to the complete saturation of absorbent by BPA. Besides biosorption, the participation of intracellular and/or mycelium-associated enzymes in EDC biodegradation cannot be excluded. Therefore, more comprehensive studies answering the contribution of these enzymes should be performed. Until now, the main role as an alternative oxyreductase to LMEs has been assigned to intracellular cytochrome P450 (Marco-Urrea et al. 2006). This was confirmed by Wang et al. (2013), who analyzed loss of BPA in nonligninolytic conditions with *Phanerochaete sordida.* Weekly treatment showed 80% BPA reduction, while the use of cytochrome P450 inhibitor decreased the degradation efficiency to under 40%. On the other hand, the interplay of intracellular cytochrome P450 and LMEs may strongly influence EDC elimination, although the entire mechanism still remains undiscovered (Haroune et al. 2017). Therefore, LMEs are considered as a main mechanism for EDC elimination by WRF.

Each WRF can secrete a distinct type of LME depending on the species or even strain (Torres et al. 2003; Wesenberg et al. 2003; Levin et al. 2004; Yang et al. 2013b; Kinnunen et al. 2016). The enzymatic pathways of living WRF undergo the control of gene promoters, which are stimulated by an appropriate environmental factor (Suetomi et al. 2015; Toyokawa et al. 2016; Daly et al. 2020). The triggering factor for LMEs is primarily the balance of nitrogen and carbon in the medium. A high carbon/nitrogen ratio in the environment enhances the expression of enzymatic genes similar to the presence of phenolic compounds, improving WRF efficiency in the removal of contaminants (Keyser et al. 1978; Soares et al. 2005). On the other hand, the lack of sufficient trigger affects the activity of LME synthesis pathways, significantly lowering the EDC elimination rate (Janusz et al. 2013). *T. versicolor* has been the object of most studies, due to its proven high efficiency in EDC removal. The vast majority of these fungal strains secrete up to three extracellular enzymes involved in EDC decomposition (Bending et al. 2002; Takamiya et al. 2008). It can be noted from Table 2 that *T. versicolor* reached a substantial reduction (> 80%) for most tested EDC (Llorca et al. 2017; Pezzella et al. 2017; Brazkova 2019) and up to 100% for BPA (Pezzella et al. 2017). However, the remaining *Pleurotus ostreatus* and *P. chrysosporium* species, despite having a different combination of LMEs, also achieved high removal rates from 60 to over 90% (Pezzella et al. 2017; Křesinová et al. 2018). Unfortunately, due to different culture conditions and various incubation times, the presented data is hard to compare.

Despite the high productivity of the WRF on a laboratory scale under sterile and controlled conditions, such results do not provide much knowledge about fungal activity and their capacity for mycoremediation in highly variable wastewater conditions (Accinelli et al. 2010; Strong 2010; Anastasi et al. 2011; Ntougias et al. 2012; Zhang and Geißen 2012; Cruz-Morató et al. 2014). Fungi have to confront autochthonous organisms as well as multiple microcontaminants at low concentrations. Therefore, intensified research in nonsterile conditions has recently been conducted, with a view to their future industrial application (Blánquez et al. 2008; Lu et al. 2009; Cruz-Morató et al. 2013, 2014; Badia-Fabregat et al. 2015). Nonetheless, this approach faces several limitations. It has been found that the microflora naturally existing in wastewater interfere to some extent with the decomposition processes undertaken by WRF (Svobodová and Novotný 2018). Bacteria compete with fungi for nutrients and carbon sources, influencing fungal growth and synthesis of extracellular enzymes. On the other hand, bacteria decompose substances harmful to the WRF and enhance the level of nitrogen required for fungal growth (Válková et al. 2017; Mir-Tutusaus et al. 2018). In order to reduce the competition between bacteria and fungi, various strategies are applied to ensure that the culture conditions are favorable for fungi. One method is to adjust the acidic pH, optimal for fungi (Libra et al. 2003). Low pH will suppress the growth of bacteria that prefer a neutral environment, thus increasing WRF activity. However, such an approach of supporting fungal growth does not work for a long period because the bacteria are capable of adapting to acidic conditions (Mir-Tutusaus et al. 2018). Moreover, too acidic pH could result in a decrease of enzyme secretion by the WRF. Another solution implies the replacement of existing fungal biomass during degradation, due to its aging over time. The access of young mycelium allows the degradation time to be extended, also increasing the activity of the WRF (Blánquez et al. 2006; Dhouib et al. 2006; Badia-fabregat et al. 2017). Attempts have also been made to restrict the access of nitrogen to the medium, causing limited bacterial growth, though it is effective just at the beginning of the degradation since during the process, the bacteria start to absorb nitrogen from the fungi (Libra et al. 2003; Asif et al. 2017; Svobodová and Novotný 2018). This problem may be overcome by the application of extracted LMEs. Compared to the whole WRF cell, isolated enzymes are more specific to the degraded xenobiotic as well as capable of operating across a wide range of environmental conditions, thus simplifying the control of the entire process (Gassara et al. 2013; Becker et al. 2017; Falade et al. 2017). Nevertheless, the enzymes remain less efficient in degradation than the WRF due to the synergic interactions between the extracellular enzymes and mycelium (Yang et al. 2013a). In addition, fungi can secrete low molecular weight redox mediators, which can expand the range of degradable compounds (Abdel-Hamid et al. 2013; Asif et al. 2017). The next issue related to the application of enzymes includes high production and purification costs, as well as instability and no possibility of reuse (Gassara et al. 2013; Bilal et al. 2017a; Pezzella et al. 2017; Voběrková et al. 2018). Therefore, it is becoming increasingly common to implement methods of enzyme immobilization. They are based on linking the catalyst with the carrier in order to keep it in limited space and maintain its structure (Voběrková et al. 2018). The carrier should feature no toxicity, easy accessibility, and strong biological integrity with the enzyme. As the particle creates bonding with the enzyme, its structure and properties have a significant influence on the enzymatic activity of the immobilized catalyst. Both organic polymers (cellulose, starch, chitin, chitosan, silica alginate) and chemically synthesized inorganic molecules are used in the immobilization process (Al-Adhami et al. 2002; Wang et al. 2011; Kampmann et al. 2014; Verma et al. 2020). The organic ones, owing to their natural source, exhibit enhanced biological compatibility toward the enzyme. However, nowadays, nonorganic particles are gaining increasing interest (Acevedo et al. 2010; Hou et al. 2014; Ji et al. 2017). The advantage of synthetic materials is their great stiffness and highly specific surface zone, which can be easily modified through suitable functional groups according to the requirements of the situation (Barcelos et al. 2016). The catalyst can also be stabilized without supporting carrier through the construction of cross-linked enzyme conglomerates (Asgher et al. 2014). Immobilization significantly increases the stability of the enzyme thereby improving resistance to chemical and thermal denaturation. As a result, production costs are reduced due to the regenerative potential of the enzyme and the possibility of reuse (Boer 2018; Voběrková et al. 2018). Moreover, reactions involving immobilized enzymes take place in a broad spectrum of environmental conditions (Asgher et al. 2014). Since the late nineteenth century, as research on enzyme immobilization has progressed, multiple diverse methods have been developed. A distinction can be made between physical (adsorption, entrapment) and chemical (covalent bonding, cross-linking) methods (Li et al. 2012; Kim et al. 2016; Wu et al. 2018a). Physical methods do not require additional reagents and show simplicity, though the link between the carrier and the enzyme remains weak. These are mainly hydrogen bonds, hydrophobic interactions, or van der Waals forces. By contrast, in chemical methods, a stronger covalent bond is formed between the molecules. Unfortunately, the strength of the connection creates the risk of interfering with the enzyme activity (Voběrková et al. 2018; Bilal et al. 2019). Among the well-known methods of immobilization, frequently used are cross-linking, encapsulation, entrapment, or covalent bonding (Voběrková et al. 2018). So far, it is considered that the most effective technique is covalent linking, in which the enzyme is strongly attached to the carrier by covalent bonds (Gasser et al. 2014; Zhu et al. 2020). Due to the possibility of forming multiple solid connections, the stability and activity of the immobilized enzyme increases significantly. Cross-linking appears to be an equally efficient solution owing to the high stability and restoration capacity of the catalyst, as well as the economic advantage of industrial use. Additionally, this method enables two or more proteins to be immobilized in one aggregate, allowing many independent degradation processes to be conducted (Guisan 2013; Asgher et al. 2014; Bilal et al. 2017b; Voběrková et al. 2018). The choice of a suitable method is essential for the immobilization process as it determines the subsequent activity of the enzyme along with the properties of the aggregate, whereas there is no universal solution for each protein (Mohamad et al. 2015; Voběrková et al. 2018). Table 3 presents the results of the degradation efficiency of various EDCs by selected immobilized enzymes obtained by differing techniques. The majority of performed studies are focused on immobilized laccase due to its prevalence among WRF, as well as its versatility enabling numerous technological applications (Asgher et al. 2014). Research on immobilized laccase has shown a very high degree of EDC reduction (> 85%) (Gamallo et al. 2018; Zdarta et al. 2018; Bilal et al. 2019; Maryskova et al. 2019), which reached even 100% in the case of laccase from *P. ostreatus* (Brugnari et al. 2018). Nevertheless, in single studies using universal and manganese peroxidase, a sufficient degradation rate, exceeding 95%, has also been achieved (Taboada-Puig et al. 2011; Bilal et al. 2017b). In addition, an experiment involving several LMEs proved to be equally productive, with a 90% decrease in BPA (Gassara et al. 2013). Promisingly, in all mentioned studies involving immobilized enzymes (Table 3), there were observed comparable or improved degradation results as whole-cell WRF (Table 2) in a significantly shorter incubation time not exceeding 24 h. Thus, the immobilized enzymes exhibit the potential for future industrial use upon improved optimization and reduced production costs.

**Table 3:** Degradation efficiency (%) of various EDC achieved by selected immobilized enzymes by using distinct immobilization strategies

Enzyme type	Fungal species	Immobilization type	Incubation time	EDC	Initial concentration (mg/L)	Removal efficiency (%)	References
Laccase	*T. versicolor*	*H. communis* spongin-based scaffolds	24 h	Bisphenol A	2	96	Zdarta et al. (2018)
			24 h	Bisphenol S	2	53	
			24 h	Bisphenol F	2	95	
		Nanofiber membrane	24 h	Bisphenol A	11.4	88	Maryskova et al. (2019)
		Cross-linked chitosan beads	150 min	Bisphenol A	10	> 99	Bilal et al. (2019)
	*M. thermophila*	Silica nanoparticles	24 h	Bisphenol A	10	> 94	Gamallo et al. (2018)
	*P. ostreatus*	MANAE-agarose	60 min	Bisphenol A	100	100	Brugnari et al. (2018)
Manganese peroxidase	*G. lucidum*	Cross-linked enzyme aggregates (CLEA®s)	150 min	Nonylphenol	5	96	Bilal et al. (2017a)
Versatile peroxidase with glucose oxidase	*B. adusta*	Cross-linked enzyme aggregates (CLEA®s)	10 min	Bisphenol A	10	95.7	Taboada-Puig et al. (2011)
			10 min	Nonylphenol	10	100	
Manganese peroxidase, lignin peroxidase, and laccase	*P. chrysosporium*	Encapsulation in polyacrylamide (PA) microgel	8 h	Bisphenol A	10	90	Gassara et al. (2013)

## Conclusions

EDC are a global problem in the environmental and health field. These compounds are constantly used in many production processes, hence negatively affecting human and animal health. Research carried out so far has shown that usage of WRF is a promising alternative for traditional wastewater treatment plants (WWTP) using activated sludge allowing for EDC removal from water. Despite the many advantages of WRF application, some challenges before using this technique on an industrial scale need to be solved. Actual WWTP are not designed for the new technology, while the adaptation is very expensive. Furthermore, regulation of the enzyme pathway in EDC degradation by WRF requires better understanding. Additionally, more comprehensive experiments should be performed on real wastewater aimed at gaining better insight into possible use of WRF in natural conditions, while prior studies have explored the degradation effectiveness of xenobiotics by specific LMEs on the laboratory scale. These studies recognized immobilized enzymes as having the greatest potential for industrial-scale use, so further tests should be undertaken in this direction.
